# Boltzmann Configurational Entropy Revisited in the Framework of Generalized Statistical Mechanics

**DOI:** 10.3390/e24020140

**Published:** 2022-01-18

**Authors:** Antonio Maria Scarfone

**Affiliations:** Istituto dei Sistemi Complessi (ISC-CNR), c/o Dipartimento di Scienza Applicata e Tecnologia del Politecnico di Torino, Corso Duca degli Abruzzi 24, 10129 Torino, Italy; antoniomaria.scarfone@cnr.it

**Keywords:** configurational entropy, (*κ*, *r*)-entropy, (*κ*, *r*)-multinomial expansion, Sharma-Taneja-Mittal entropy, 05.90.+m, 05.20.-y, 02.70.Rr

## Abstract

As known, a method to introduce non-conventional statistics may be realized by modifying the number of possible combinations to put particles in a collection of single-particle states. In this paper, we assume that the weight factor of the possible configurations of a system of interacting particles can be obtained by generalizing opportunely the combinatorics, according to a certain analytical function $f_{\left\{\pi \right\}}\left(n\right)$ of the actual number of particles present in every energy level. Following this approach, the configurational Boltzmann entropy is revisited in a very general manner starting from a continuous deformation of the multinomial coefficients depending on a set of deformation parameters {π}. It is shown that, when $f_{\left\{\pi \right\}}\left(n\right)$ is related to the solutions of a simple linear difference–differential equation, the emerging entropy is a scaled version, in the occupational number representation, of the entropy of degree (κ,r) known, in the framework of the information theory, as Sharma–Taneja–Mittal entropic form.

## 1. Introduction

Boltzmann entropy of a microcanonical ensemble is a measure of the number of possible microstates W[n] of a *n*-identical particles system, for a given macrostate characterized, in the simplest case, by the total particle number *n* and the total energy *E*. Sometimes called configurational entropy, Boltzmann entropy is given by the logarithm of the microstates number

(1) $$\begin{array}{c} S^{B}\left[n\right]=\ln\left(W[n]\right)\;. \end{array}$$

From elementary arguments in combinatorial analysis, it turns out that W(n) is given by the multinomial coefficient:

(2) $$\begin{array}{c} W\left[n\right]=\left[\begin{array}{c} n\\ n_{1}\;\ldots \;n_{\ell } \end{array}\right]\equiv \frac{n!}{n_{1}!\;n_{2}!\ldots n_{\ell }!}\;, \end{array}$$

constrained by the conditions $n=\sum_{i}n_{i}$ and $E=\sum_{i}n_{i}\;\epsilon_{i}$, where $n=\{n_{i}\}$ is the particle number distribution among the available distinguishable states, characterized by the energy levels $\epsilon_{i}$, with i=1,…,ℓ, being $n_{i}$ the particles number occurring in the *i*th level.

As known, (1) can be related to Shannon entropy,

(3) $$\begin{array}{c} S^{S}\left[p\right]=-\sum_{i=1}^{\ell }p_{i}\;\ln\left(p_{i}\right)\;, \end{array}$$

whose expression is derived in the context of the information theory. This follows by introducing the discrete probability distribution $p=\{p_{i}\}$ to finding a particular ensemble configuration, consistent with the given energy *E* of the system. In the limit of large *n*, such that $n_{i}=n\;p_{i}$, it is straightforward to obtain the relation:

(4) $$\begin{array}{c} \frac{1}{n}S^{B}\left[n\right]\approx S^{S}\left[p\right]\;. \end{array}$$

According to the maximal entropy principle, the most probable particle configuration is the one that maximizes the configurational entropy under the appropriate constraints given, in this case, by *n* and *E*. As known, such statistics is characterized by an exponential tail that is typical of the Boltzmann–Gibbs distribution.

However, deviations from the Boltzmann–Gibbs statistics are observed in quasi-particle excitations occurring in several condensed matter systems. Therefore, the study of physical systems obeying non-conventional statistics is one of the most investigated topics of contemporary statistical physics [1,2,3,4,5,6,7]. Their interest goes from the theoretical foundation of generalized statistical mechanics [8,9,10,11,12], to high-temperature gas [13] and high-$T_{c}$ superconductivity [14], Laughlin particle with a fractional charge related to fractional quantum Hall effect [15], Josephson junctions [16], and others.

A way to introduce non conventional statistics is given by modifying the number of possible ways to put particles into a collection of single-particle states. This corresponds to changing the counting rule in (2). In this way it is possible to derive several generalized statistics. Among them, we recall the Haldane-Wu statistics [17,18], semion [19], the intermediate statistics by Gentile [20], ewkons [21] and the interpolating Boson-Fermion statistics [22,23,24,25].

Within this approach, in [26,27] a possible generalization of the microcanonical counting based on the *q*-deformed algebra arising in the framework of the nonextensive statistical mechanics [28] has been advanced. This mathematical structure is quite fundamental to the theoretical foundation of the *q*-generalized statistical physics as does ordinary algebra in standard statistical physics. In particular, by introducing an opportunely defined multinominal coefficient with its Stirling approximation, in [26] Authors proposed a *q*-version of (4) that links the logarithm of the *q*-multinominal coefficient with the *q*-generalized version of the entropy (3) introduced in [28] (and references therein).

Similarly, a combinatorial approach for κ-entropy introduced in [29,30] was presented in [31]. It is based on the κ-algebra [32] linked to the one-parameter deformed κ-exponential and κ-logarithmic functions. The resulting κ-generalized statistical mechanics is nowadays under examination both for its theoretical foundations and for its potential applications in physics and physical-like systems [33,34,35,36,37,38,39,40,41,42,43,44].

In this paper, we present a possible generalization of the Boltzmann counting (2), based on a certain counting rule fixed by a monotonic increasing analytical function $f_{\left\{\pi \right\}}\left(x\right)$ depending, in general, on a set of deformation parameters {π}, and reducing to the identity in a suitable limit.

Among the many possible choices, we are mainly interested in those generating functions $f_{\left\{\pi \right\}}\left(x\right)$ that lead to generalized entropic forms consistent with power-law distributions. Often, probability distribution functions observed in complex systems (like sociophysics, econophysics, biophysics and others) are plagued by the heavy tail that confers to the system an *anomalous* statistical behavior which differs significantly from that exponential characterizing the Boltzmann-Gibbs distribution. Such *anomalous* statistical proprieties are embodied, in some sense, by the associated entropic form from which the (meta)-equilibrium distribution is derived according to the already cited maximal entropy principle.

Thus, by requiring that $f_{\left\{\pi \right\}}\left(x\right)$ be related to the solutions of a simple delay equation, we obtain a two-parameters family of generalization of the Boltzmann configurational entropy (1) that, in the large *n* limit, reproduces a scaled version of entropy of degree (κ,r) introduced by Sharma and Taneja [45] and Mittal [46]. In particular, the *q*-case and the κ-case are derived from the general discussion in the opportune limit of the deformation parameters.

As known, entropy is also a fundamental quantity used in the formulation of the second law of thermodynamics. According to the Boltzmann H-theorem, it governs the approach toward the equilibrium configuration of a system plague by dissipative processes like, for instance, diffusion or heat transfer [47]. Indeed, generalized entropic form might likewise be applied to investigate such physical situations in a more efficient manner to account for sub-diffusive or super-diffusive processes [48] as well as non-Fourier heat conduction [49], anomalous entropy production [50], and other.

The plane of the paper is as follows. In the next Section 2, we derive the generalized-multinomial coefficient and the corresponding configurational entropy by introducing an suitable counting rule for the occupational number in the framework of a generalized-deformed algebra modeled by a function $f_{\left\{\pi \right\}}\left(x\right)$. In Section 3, we explore the asymptotic behavior of the emerging entropic form by assuming that $f_{\left\{\pi \right\}}\left(x\right)$ is related to the solution of a simple differential-difference equation and obtaining, in this case, the scaled version of the entropy of degree (κ,r). For this two-parameters case, in Section 4 we generalize relation (4) and then, in Section 5, we specialize this asymptotic relation to the case of one-deformation parametric entropies like the *q*-entropy and the κ-entropy. Finally, Section 6 reports some concluding remarks while a technical appendix closes this work.

## 2. Occupational Counting in the Generalized Statistics

Let us consider a microcanonical system of *n* interacting particles with total energy *E*, endowed by *ℓ* distinguishable energy levels $\epsilon_{i}$. Suppose that the effects of the interactions among the particles give rise to a kind of correlation that may be taken into account by changing opportunely the computing of the number of possible microstates W[n] corresponding to the given macrostate (E,n), according to a certain prescription. This can be realized by introducing a continuous function $f_{\left\{\pi \right\}}\left(x\right)$, depending on a set of deformation parameters $\left\{\pi \right\}=\left\{\pi_{1},\;\pi_{2},\;\ldots \right\}$, that *control* the effects of the correlations among the particles, such that the elementary combinatorial calculus changes according to:

(5) $$\begin{array}{c} n!\to f_{\left\{\pi \right\}}\left(n\right)!=f_{\left\{\pi \right\}}\left(n\right)\;f_{\left\{\pi \right\}}\left(n-1\right)\;\ldots \;f_{\left\{\pi \right\}}\left(1\right)\;. \end{array}$$

It is meant that $f_{\left\{\pi \right\}}\left(x\right)$ reduces to identity in a suitable limit $\left\{\pi \right\}\to \left\{\pi_{0}\right\}$, i.e., $f_{\left\{\pi \right\}}\left(x\right)\to f_{\left\{\pi_{0}\right\}}\left(x\right)=x$, where correlations, and hence interactions among particles, are supposed to be turning off in the same limit.

We stress that prescription (5) is not just a mere isomorphism $x\to f_{\left\{\pi \right\}}\left(x\right)$ between the real numbers which would be irrelevant for the sake of the new statistics, but rather, it represents a different manner to compute the statistical weight of a given configuration, that is, the combinatorial number of possible microscopical configurations available for a given macrostate. Clearly, it must reduced to the standard combinatorial calculus in the $\left\{\pi \right\}\to \left\{\pi_{0}\right\}$ limit, a fact that is guaranteed by the condition $f_{\left\{\pi_{0}\right\}}\left(x\right)=x$.

Rule (5) is well known within the basic calculus [51] at the foundation of quons algebra and quons statistics, where the basic factorial is defined in [n]!=[n][n−1]…[1], that is just (5) with $\left\{\pi \right\}\equiv \tilde{q}$ and $f_{\tilde{q}}\left(n\right)=\left[n\right]$ for the basic number $\left[n\right]=\frac{{\tilde{q}}^{n}-1}{\tilde{q}-1}$.

Reasonably, we require that $f_{\left\{\pi \right\}}\left(x\right)$ be a strictly monotone increasing positive function defined at least on ${ℜ}^{+}$, with $f_{\left\{\pi \right\}}\left(0\right)=0$. Starting from the function $f_{\left\{\pi \right\}}\left(x\right)$ we can introduce a commutative semigroup on ${ℜ}^{+}$, i.e., an algebraic structure $A\equiv ({ℜ}^{+};\;⊗)$ equipped with a binary operation that is commutative x⊗y=y⊗x and associative x⊗(y⊗z)=(x⊗y)⊗z, for any $x,\;y,\;z\in {ℜ}^{+}$, where the {π}-product and its inverse operation the {π}-division are defined in:

(6) $$\begin{array}{ccc} & & x⊗y=f_{\left\{\pi \right\}}^{-1}\left(f_{\left\{\pi \right\}}\left(x\right)\times f_{\left\{\pi \right\}}\left(y\right)\right)\;, \end{array}$$

(7) $$\begin{array}{ccc} & & x⊘y=f_{\left\{\pi \right\}}^{-1}\left(f_{\left\{\pi \right\}}\left(x\right)/f_{\left\{\pi \right\}}\left(y\right)\right)\;, \end{array}$$

and reduces to the standard product and division operations in the $\left\{\pi \right\}\to \left\{\pi_{0}\right\}$ limit.

By iteration, we can also introduce the {π}-power $x^{⊗n}$ on ${ℜ}^{+}$, which reduces to the ordinary operation $x^{n}$ in the $\left\{\pi \right\}\to \left\{\pi_{0}\right\}$ limit, according to:

(8) $$\begin{array}{c} x^{⊗n}=f_{\left\{\pi \right\}}^{-1}\left(\overset{\;n-\mathrm{times}}{\overbrace{f_{\left\{\pi \right\}}\left(x\right)\cdot \;\ldots \cdot f_{\left\{\pi \right\}}\left(x\right)}}\right)\;. \end{array}$$

Link to the ordinary algebra can be realized by introducing the {π}-logarithm,

(9) $$\begin{array}{c} \ln_{\left\{\pi \right\}}\left(x\right)=\ln\left(f_{\left\{\pi \right\}}\left(x\right)\right)\;, \end{array}$$

that satisfies the relations

(10) $$\begin{array}{ccc} & & \ln_{\left\{\pi \right\}}\left(x⊗y\right)=\ln_{\left\{\pi \right\}}\left(x\right)+\ln_{\left\{\pi \right\}}\left(y\right)\;,\\ & & \ln_{\left\{\pi \right\}}\left(x⊘y\right)=\ln_{\left\{\pi \right\}}\left(x\right)-\ln_{\left\{\pi \right\}}\left(y\right)\;,\\ & & \ln_{\left\{\pi \right\}}\left(x^{⊗y}\right)=y\;\ln_{\left\{\pi \right\}}\left(x\right)\;. \end{array}$$

In the same way, the {π}-exponential is introduced as:

(11) $$\begin{array}{c} \exp_{\left\{\pi \right\}}\left(x\right)=f_{\left\{\pi \right\}}^{-1}\left(\exp(x)\right)\;, \end{array}$$

and satisfies the relations:

(12) $$\begin{array}{ccc} & & \exp_{\left\{\pi \right\}}\left(x+y\right)=\exp_{\left\{\pi \right\}}\left(x\right)⊗\exp_{\left\{\pi \right\}}\left(y\right)\;,\\ & & \exp_{\left\{\pi \right\}}\left(x-y\right)=\exp_{\left\{\pi \right\}}\left(x\right)⊘\exp_{\left\{\pi \right\}}\left(y\right)\;,\\ & & \exp_{\left\{\pi \right\}}\left(x\;y\right)=\exp_{\left\{\pi \right\}}{\left(x\right)}^{⊗y}\;. \end{array}$$

Both $\ln_{\left\{\pi \right\}}\left(x\right)$ and $\exp_{\left\{\pi \right\}}\left(x\right)$ reduce to the standard functions, respectively ln(x) and exp(x), in the $\left\{\pi \right\}\to \left\{\pi_{0}\right\}$ limit.

Remark that, whenever the function $f_{\left\{\pi \right\}}\left(x\right)$ enjoys the property $f_{\left\{\pi \right\}}\left(1/x\right)=1/f_{\left\{\pi \right\}}\left(x\right)$, the generalized logarithm and exponential satisfy the relations $\ln_{\left\{\pi \right\}}\left(1/x\right)=-\ln_{\left\{\pi \right\}}\left(x\right)$ and $\exp_{\left\{\pi \right\}}\left(-x\right)=1/\exp_{\left\{\pi \right\}}\left(x\right)$ like the usual logarithm and exponential functions do. In this case

(13) $$\begin{array}{c} x⊘y=x⊗\frac{1}{y}\;. \end{array}$$

Following prescription (5) we assume that the statistical weight of an interacting particles system is modified in an *equivalent* number of microstates, according to

(14) $$\begin{array}{c} W\left[n\right]\to {\tilde{W}}_{\left\{\pi \right\}}\left[n\right]\equiv \frac{f_{\left\{\pi \right\}}\left(n\right)!}{\prod_{i=1}^{\ell }f_{\left\{\pi \right\}}\left(n_{i}\right)!}\;, \end{array}$$

which reduces to the usual Boltzmann counting (2) in the $\left\{\pi \right\}\to \left\{\pi_{0}\right\}$ limit where the system becomes an ideal ensemble of non-interacting particles.

The next step is a pillar in statistical physics and consists to introduce the configurational entropy of the system as the standard logarithm of the microstates number [52], that is

(15) $$\begin{array}{c} S_{\left\{\pi \right\}}^{B}\left[n\right]=\ln\left({\tilde{W}}_{\left\{\pi \right\}}\left[n\right]\right)\;. \end{array}$$

However, in the picture of the deformed algebra, it would be more appropriate to write this last equation throughout the {π}-logarithm rather than the ordinary one. Therefore, accounting for (9), we rewrite entropy (15) in:

(16) $$\begin{array}{c} S_{\left\{\pi \right\}}^{B}\left[n\right]=\ln_{\left\{\pi \right\}}\left(W_{\left\{\pi \right\}}\left[n\right]\right)\;, \end{array}$$

where the {π}-deformed Boltzmann counting function now is defined as

(17) $$\begin{array}{c} W_{\left\{\pi \right\}}\left[n\right]=f_{\left\{\pi \right\}}^{-1}\left({\tilde{W}}_{\left\{\pi \right\}}\left[n\right]\right)\;. \end{array}$$

We stress once again that (16) corresponds to the orthodox definition of microcanonical entropy (1) of a system with ${\tilde{W}}_{\left\{\pi \right\}}\left[n\right]$*equivalent* number of microstates, although it is written, for sake of formalism, by means of the {π}-deformed logarithm.

Furthermore, by recalling the definition of the {π}-product, we can rewrite (17) in:

(18) $$\begin{array}{c} W_{\left\{\pi \right\}}\left[n\right]=n{!}_{\left\{\pi \right\}}⊘\left(\prod_{i=1}^{\ell }{}^{⊗}n_{i}{!}_{\left\{\pi \right\}}\right)\;, \end{array}$$

where $\prod^{⊗}$ denotes a sequence of {π}-products and $n{!}_{\left\{\pi \right\}}$, given in

(19) $$\begin{array}{c} n{!}_{\left\{\pi \right\}}=f_{\left\{\pi \right\}}^{-1}\left(f_{\left\{\pi \right\}}\left(n_{j}\right)!\right)\equiv n⊗\left(n-1\right)⊗\ldots ⊗1\;, \end{array}$$

is the generalized factorial whose definition has been advanced for the first time in [26] in the formalism of the *q*-deformed algebra.

## 3. Boltzmann Entropy of Degree (κ,r)

To go one step further, we use (10) to expand the {π}-logarithm of $n{!}_{\left\{\pi \right\}}$ in a manner that mimics the same relation occurring in the standard algebra, that is

(20) $$\begin{array}{c} \ln_{\left\{\pi \right\}}\left(n{!}_{\left\{\pi \right\}}\right)=\sum_{i=1}^{n}\ln_{\left\{\pi \right\}}\left(i\right)\approx \int_{0}^{n}\ln_{\left\{\pi \right\}}\left(x\right)\;dx\;, \end{array}$$

where, following the approach proposed in [26], we approximate the summation for large *n* by an integration.

By introducing the function:

(21) $$\begin{array}{c} y\left(x\right)=\ln_{\left\{\pi \right\}}\left(\exp(x-\delta )\right)\;, \end{array}$$

it is shown in Appendix A, that whenever y(x) is a solution of the following delay equation

(22) $$\begin{array}{c} \frac{d\;y(x)}{dx}=\lambda \;y\left(x+\delta \right)-y\left(x\right)\;, \end{array}$$

with λ and δ constants to be determined, (20) can be straightforward integrated in:

(23) $$\begin{array}{c} \ln_{\left\{\pi \right\}}\left(n{!}_{\left\{\pi \right\}}\right)\approx \frac{n}{\lambda_{\left\{\pi \right\}}}\ln_{\left\{\pi \right\}}\left(\frac{n}{\delta_{\left\{\pi \right\}}}\right)\;, \end{array}$$

where $\lambda_{\left\{\pi \right\}}$ and $\delta_{\left\{\pi \right\}}$ are still constants. Equation (23) represents a generalization of the well known Stirling approximation, holding for large *n*.

Under certain assumptions (see Appendix A), the space of the deformation parameters reduces to a two-dimensional region R={−|κ|<r<|κ|, if 0≤|κ|<1/2 and |κ|−1<r<1−|κ|, if 1/2≤|κ|<1}, with {π}≡{κ,r}, and the generalized logarithm takes the explicit expression:

(24) $$\begin{array}{c} \ln_{\kappa ,r}\left(x\right)=\{\begin{array}{c} x^{r}\;\frac{x^{\kappa }-x^{-\kappa }}{2\;\kappa }\;,\;\mathrm{for}\;0<\kappa <1\;,\\ \ln(x)\;,\;\mathrm{for}\;\kappa =0, \end{array} \end{array}$$

that is a continuous, monotonic, increasing and concave function for $x\in {ℜ}^{+}\to ℜ$, with $\ln_{\kappa ,r}\left(x\right)=-\ln_{\kappa ,-r}\left(1/x\right)$. The two constants $\lambda_{\left\{\pi \right\}}\equiv \lambda_{\kappa ,r}$ and $\delta_{\left\{\pi \right\}}\equiv e_{\kappa ,r}$, given by:

(25) $$\begin{array}{ccc} & & \lambda_{\kappa ,r}=\{\begin{array}{c} \frac{{\left(1+r-\kappa \right)}^{(r+\kappa )/2\;\kappa }}{{\left(1+r+\kappa \right)}^{(r-\kappa )/2\;\kappa }}\;,\;\mathrm{for}\;0<\kappa <1\;,\\ 1\;,\;\mathrm{for}\;\kappa =0\;, \end{array} \end{array}$$

(26) $$\begin{array}{ccc} & & e_{\kappa ,r}=\{\begin{array}{c} {\left(\frac{1+r+\kappa }{1+r-\kappa }\right)}^{1/2\;\kappa }\;,\;\mathrm{for}\;0<\kappa <1\;,\\ e\;,\;\mathrm{for}\;\kappa =0\;, \end{array} \end{array}$$

which are well defined in R and are related in $\ln_{\kappa ,r}\left(e_{\kappa ,r}\right)=1/\lambda_{\kappa ,r}$. These constants reduce in the {κ,r}→(0,0) limit to $\lambda_{\kappa ,r}\to \lambda_{0,0}=1$ and $e_{\kappa ,r}\to e_{0,0}=e$, the Neperian number, as well as, in the same limit, $\ln_{\kappa ,r}\left(x\right)\to \ln_{0,0}\left(x\right)=\ln\left(x\right)$.

Therefore, in the present case, the configurational entropy (16) becomes

(27) $$\begin{array}{c} S_{\kappa ,r}^{B}\left[n\right]=\ln_{\kappa ,r}\left(n{!}_{\kappa ,r}\right)-\sum_{i=1}^{\ell }\;\ln_{\kappa ,r}\left(n_{i}{!}_{\kappa ,r}\right)\;, \end{array}$$

and, by using the asymptotic approximation (23), it can be rewritten in:

(28) $$\begin{array}{c} S_{\kappa ,r}^{B}\left[n\right]=-\frac{1}{\lambda_{\kappa ,r}}\sum_{i=1}^{\ell }n_{i}\;\ln_{\kappa ,r}\left(\frac{n_{i}}{e_{\kappa ,r}}\right)+S_{\kappa ,r}\left(n\right)\;, \end{array}$$

where

(29) $$\begin{array}{c} S_{\kappa ,r}\left(n\right)=\frac{n}{\lambda_{\kappa ,r}}\;\ln_{\kappa ,r}\left(\frac{n}{e_{\kappa ,r}}\right)\;, \end{array}$$

is a function depending only on the total particle number. It guarantees the right normalization of the entropy (28) fixed by the relation $S_{\kappa ,r}^{B}\left[\left\{n,\;0,\;0,\;\ldots \right\}\right]=0$.

Remark that, due to the definition of $e_{\kappa ,r}$, entropy (28) diverges in the r→κ−1 limit whenever 1/2≤|κ|<1.

From (28), by using (10), the asymptotic expression of the Boltzmann counting function (18) can be written in:

(30) $$\begin{array}{c} W_{\kappa ,r}\left[n\right]\approx {\left(\frac{n}{e_{\kappa ,r}}\right)}^{⊗\frac{n}{\lambda_{\kappa ,r}}}⊘\left(\prod_{i=1}^{\ell }{}^{⊗}{\left(\frac{n_{i}}{e_{\kappa ,r}}\right)}^{⊗\frac{n_{i}}{\lambda_{\kappa ,r}}}\right)\;, \end{array}$$

which mimics the corresponding function for a system of non interacting particles that is recovered in the (κ,r)→(0,0) limit. In particular, (30) says that the statistical weight of a system of interacting particles can be modeled like that of a free particles system in a way such that the effects of correlations are embodied and taken into account through the underlying generalized algebra.

## 4. Connection with the Shannon Entropy of Degree (κ,r)

In the framework of the information theory, an entropic form similar to (28) is known as entropy of degree (r,s). It was introduced firstly, about fifty years ago, in the form [45,46]:

(31) $$\begin{array}{c} S_{r,\;s}\left[p\right]=\frac{1}{2^{1-r}-2^{1-s}}\sum_{i=1}^{\ell }\left(p_{i}^{r}-p_{i}^{s}\right)\;, \end{array}$$

and then rediscovery in the statistical mechanics framework [53], in the form:

(32) $$\begin{array}{c} S_{\kappa ,r}\left[p\right]=\sum_{i=1}^{\ell }\frac{p_{i}^{1+r-\kappa }-p_{i}^{1+r+\kappa }}{2\;\kappa }\equiv -\sum_{i=1}^{\ell }p_{i}\;\ln_{\kappa ,r}\left(p_{i}\right)\;. \end{array}$$

Boltzmann expression (28), in the occupation number representation, is a scaled version of entropy (32) in the probability representation. The former, named para-entropy of degree (κ,r) [54], can be put in relation with the latter by generalizing opportunely relation (4).

Firstly, let us recall the composition law of the (κ,r)-logarithm:

(33) $$\begin{array}{c} \ln_{\kappa ,r}\left(x\;y\right)=\ln_{\kappa ,r}\left(x\right)\;u_{\kappa ,r}\left(y\right)+u_{\kappa ,r}\left(x\right)\;\ln_{\kappa ,r}\left(y\right)\;, \end{array}$$

where the function $u_{\kappa ,r}\left(x\right)$, given in

(34) $$\begin{array}{c} u_{\kappa ,r}\left(x\right)=\{\begin{array}{c} x^{r}\;\frac{x^{\kappa }+x^{-\kappa }}{2}\;,\;\mathrm{for}\;0<\kappa <1\;,\\ 1\;,\;\mathrm{for}\;\kappa =0 \end{array} \end{array}$$

is, in the parameter region R, a continuous function for $x\in {ℜ}^{+}\to {ℜ}^{+}$, with $u_{\kappa ,r}\left(x\right)=u_{\kappa ,-r}\left(1/x\right)$, $u_{\kappa ,r}\left(e_{\kappa ,r}\right)=\left(1+r\right)/\lambda_{\kappa ,r}$ and reduces to $u_{0,0}\left(x\right)=1$ in the (κ,r)→(0,0) limit.

By introducing the probability of the occupation number according to $n_{i}=n\;p_{i}$ and using (33) in $\ln_{\kappa ,r}\left(n_{i}/e_{\kappa ,r}\right)=\ln_{\kappa ,r}\left(n\;p_{i}/e_{\kappa ,r}\right)$, we have

(35) $$\begin{array}{ccc} & & -\frac{1}{\lambda_{\kappa ,r}}\sum_{i=1}^{\ell }n\;p_{i}\;\ln_{\kappa ,r}\left(\frac{n\;p_{i}}{e_{\kappa ,r}}\right)\\ & = & -\frac{n}{\lambda_{\kappa ,r}}\sum_{i=1}^{\ell }p_{i}\left(\ln_{\kappa ,r}\left(\frac{n}{e_{\kappa ,r}}\right)\;u_{\kappa ,r}\left(p_{i}\right)+u_{\kappa ,r}\left(\frac{n}{e_{\kappa ,r}}\right)\;\ln_{\kappa ,r}\left(p_{i}\right)\right)\\ & = & -\frac{n}{\lambda_{\kappa ,r}}\;\ln_{\kappa ,r}\left(\frac{n}{e_{\kappa ,r}}\right)\sum_{i=1}^{\ell }p_{i}\;u_{\kappa ,r}\left(p_{i}\right)-\frac{n}{\lambda_{\kappa ,r}}\;u\left(\frac{n}{e_{\kappa ,r}}\right)\sum_{i=1}^{\ell }p_{i}\;\ln_{\kappa ,r}\left(p_{i}\right)\;. \end{array}$$

It is now useful to introduce the new functional,

(36) $$\begin{array}{c} I_{\kappa ,r}\left[p\right]=\sum_{i=1}^{\ell }p_{i}\;u_{\kappa ,r}\left(p_{i}\right)\;, \end{array}$$

that plays a special role in the present theory being strictly related to entropy $S_{\kappa ,r}\left[p\right]$ [35,55]. It is defined as the linear average of $u_{\kappa ,r}\left(x\right)$ as well as entropy being the linear average of $\ln_{\kappa ,r}\left(x\right)$. Furthermore, in the same spirit of (29), let us define the quantity:

(37) $$\begin{array}{c} N_{\kappa ,r}\left(n\right)=\frac{n}{\lambda_{\kappa ,r}}\;u_{\kappa ,r}\left(\frac{n}{e_{\kappa ,r}}\right)\;, \end{array}$$

which is a function depending only on the total particle number. In particular, in the (κ,r)→(0,0) limit, these functions reduces, respectively, to the identity $I_{0,0}\left[p\right]=\sum_{i}p_{i}=1$, and to the total particle number $N_{0,0}\left(n\right)=n$.

In this way, recalling the entropy definition (28), relation (35) can be rewritten in:

(38) $$\begin{array}{c} \frac{1}{N_{\kappa ,r}\left(n\right)}\;S_{\kappa ,r}^{B}\left[n\right]\approx S_{\kappa ,r}^{S}\left[p\right]-\frac{S_{\kappa ,r}\left(n\right)}{N_{\kappa ,r}\left(n\right)}\;\left(I_{\kappa ,r}\left[p\right]-1\right)\;, \end{array}$$

which is the generalized version of (4), which is reobtained in the opportune limit.

Furthermore, since this relation holds in the large *n* limit, whenever (κ,r)≠(0,0), and using the following straightforward asymptotic relations:

(39) $$\begin{array}{c} S_{\kappa ,r}\left(n\right)\approx \frac{n^{1+r+\kappa }}{2\;\kappa \;(1+r+\kappa )}\;,\;N_{\kappa ,r}\left(n\right)\approx \frac{n^{1+r+\kappa }}{2\;(1+r+\kappa )}\;, \end{array}$$

Equation (38) can be rewritten in the simplest form:

(40) $$\begin{array}{c} \frac{2\;(1+r+\kappa )}{n^{1+r+\kappa }}\;S_{\kappa ,r}^{B}\left[n\right]\approx S_{\kappa ,r}^{S}\left[p\right]-\frac{1}{\kappa }\;\left(I_{\kappa ,r}\left[p\right]-1\right)\;. \end{array}$$

It is worth observing that the configurational entropy $S_{\kappa ,r}^{B}\left[n\right]$ is no more proportional to the probability entropy $S_{\kappa ,r}^{S}\left[p\right]$ like occurs in the simple case (4). This fact stays to indicates that not all the information carried by the configurational entropy is now contained also in the probability entropy and a further statistical quantity, represented by the function $I_{\kappa ,r}\left[p\right]$, is, in general, required. This happens whenever the system under investigation is enough complex to be described by a generalized entropic form instead of the Shannon–Boltzmann–Gibbs one that, as widely established, is able to capture the statistical behavior of not interacting or weakly interacting systems.

## 5. Some Particular Cases

Sharma–Taneja–Mittal entropic form (32) contains some special cases characterized by one-parameters deformation. Among these, the κ-entropy [29] and the *q*-entropy [28] have been widely employed in the study of statistical and statistical-mechanics behavior observed in different physics and physical-like systems characterized by the presence of complex mechanisms. In the sequel, we briefly specialize the result obtained in the previous sections to these two generalized entropies to make contact with the existent literature.

### 5.1. κ-Deformed Boltzmann Entropy

The κ-entropy, derived starting from argumentations based on the special relativity theory [29,30], follows from (32) by posing r=0

(41) $$\begin{array}{c} S_{\kappa }\left[p\right]=-\sum_{i=1}^{\ell }\frac{p_{i}^{1+\kappa }-p_{i}^{1-\kappa }}{2\;\kappa }\;. \end{array}$$

In the spirit of the κ-algebra, underlying to the generalized statistical mechanics founded on the κ-entropy, we define the function

(42) $$\begin{array}{c} f_{\kappa }\left(x\right)=\exp\left(\frac{1}{\kappa }\;\sinh\left(\kappa \;\ln(x)\right)\right)\;, \end{array}$$

so that, the explicit expressions of the κ-logarithm $\ln_{\kappa }\left(x\right)=-\ln_{\kappa }\left(1/x\right)$ and κ-exponential $\exp_{\kappa }\left(x\right)=1/\exp_{\kappa }\left(-x\right)$ are

(43) $$\begin{array}{ccc} & & \ln_{\kappa }\left(x\right)=\{\begin{array}{c} \frac{x^{\kappa }-x^{-\kappa }}{2\;\kappa }\;,\;\mathrm{for}\;0<\kappa <1\;,\\ \ln(x)\;,\;\mathrm{for}\;\kappa =0\;, \end{array} \end{array}$$

(44) $$\begin{array}{ccc} & & \exp_{\kappa }\left(x\right)=\{\begin{array}{c} {\left(\kappa \;x+\sqrt{1+\kappa^{2}\;x^{2}}\right)}^{1/\kappa }\;,\;\mathrm{for}\;0<\kappa <1\;,\\ \exp(x)\;,\;\mathrm{for}\;\kappa =0\;. \end{array} \end{array}$$

In this case, the generalized product reads [32]:

(45) $$\begin{array}{c} x⊗y=\exp\left(\frac{1}{\kappa }\;\mathrm{arcsinh}\left(\sinh\left(\kappa \;\ln(x)\right)+\sinh\left(\kappa \;\ln(y)\right)\right)\right)\;, \end{array}$$

whilst, the asymptotic approximation of $\ln_{\kappa }\left(n{!}_{\kappa }\right)$ obtained from (23) is given by

(46) $$\begin{array}{c} \ln_{\kappa }\left(n{!}_{\kappa }\right)\approx \int_{0}^{n}\ln_{\kappa }\left(x\right)\;dx=\frac{n}{\lambda_{\kappa }}\;\ln_{\kappa }\left(\frac{n}{e_{\kappa }}\right)\;, \end{array}$$

where the two constants $\lambda_{\kappa }=\sqrt{1-\kappa^{2}}$ and $e_{\kappa }={\left[(1+\kappa )/(1-\kappa )\right]}^{\frac{1}{2\;\kappa }}$ are well defined in the range |κ|<1.

The κ-version of the configurational entropy (28) is

(47) $$\begin{array}{c} S_{\kappa }^{B}\left[n\right]\approx -\frac{1}{\lambda_{\kappa }}\sum_{i=1}^{\ell }n_{i}\;\ln_{\kappa }\left(\frac{n_{i}}{e_{\kappa }}\right)+S_{\kappa }\left(n\right)\;, \end{array}$$

and, by using (33) specialized to the κ-case, we can rewrite (47) in

(48) $$\begin{array}{c} S_{\kappa }^{B}\left[n\right]\approx \frac{1}{\lambda_{\kappa }^{2}}\;\left(S_{\kappa }\left[n\right]+I_{\kappa }\left[n\right]\right)+S_{\kappa }\left(n\right)\;, \end{array}$$

where

(49) $$\begin{array}{c} I_{\kappa }\left[n\right]=\sum_{i=1}^{\ell }\frac{n_{i}^{1+\kappa }+n_{i}^{1-\kappa }}{2}\;. \end{array}$$

Finally, posed $p_{i}=n_{i}/n$ in the r.h.s. of (48), we obtain the relation

(50) $$\begin{array}{c} S_{\kappa }^{B}\left[n\right]\approx \frac{n^{1+\kappa }}{2\;\kappa \;(1+\kappa )}\;\left[1-\sum_{i=1}^{\ell }{\left(\frac{n_{i}}{n}\right)}^{1+\kappa }\right]+\frac{n^{1-\kappa }}{2\;\kappa \;(1-\kappa )}\;\left[\sum_{i=1}^{\ell }{\left(\frac{n_{i}}{n}\right)}^{1-\kappa }-1\right]\;, \end{array}$$

that corresponds to (38) for r=0, and, in the limit of large *n*, whenever κ≠0, (50) can be written in the form:

(51) $$\begin{array}{c} \frac{2\;(1+|\kappa |)}{n^{1+|\kappa |}}\;S_{\kappa }^{B}\left[n\right]\approx S_{\kappa }^{S}\left[p\right]-\frac{1}{\left|\kappa \right|}\;\left(I_{\kappa }\left[p\right]-1\right)\;, \end{array}$$

corresponding to (40).

We observe that (50) has been previously obtained in [31]. In order to overcame the question related to the lost of proportionality between the configurational κ-entropy $S_{\kappa }^{B}\left[n\right]$ and the probability κ-entropy $S_{\kappa }^{S}\left[p\right]$, in [31] has been suggest to modify the l.h.s. of (50) adding a further function of a certain combinatorial capable to adsorbs the quantity $I_{\kappa }\left[n\right]/\lambda^{2}+S_{\kappa }\left(n\right)$ in (48). However, the drawback of this approach is the complication of the expression of the configurational part (the l.h.s. in (48)) in spite of the probabilistic part (the r.h.s. of (48)) and, at the same time, the introduction of a strange generalized product (in addition to the one given in (45)) to compute the new combinatorial.

### 5.2. *q*-Deformed Boltzmann Entropy

The *q*-entropy [28] follows from (32), in the so called 2−q formalism [53], by posing r=±|κ|=(1−q)/2,

(52) $$\begin{array}{c} S_{2-q}\left[p\right]=\sum_{i=1}^{\ell }\frac{p_{i}^{2-q}-p_{i}}{q-1}\;. \end{array}$$

In the present picture, it can be derived by introducing the function:

(53) $$\begin{array}{c} f_{q}\left(x\right)=\exp\left(\frac{x^{1-q}-1}{1-q}\right)\;, \end{array}$$

so that the explicit expression of the *q*-logarithm $\ln_{q}\left(x\right)=-\ln_{2-q}\left(1/x\right)$ and the *q*-exponential $\exp_{q}\left(x\right)=1/\exp_{2-q}\left(-x\right)$ are:

(54) $$\begin{array}{ccc} & & \ln_{q}\left(x\right)=\{\begin{array}{c} \frac{x^{1-q}-1}{1-q}\;,\;\mathrm{for}\;0<q<2;\;q\neq 1\;,\\ \ln(x)\;,\;\mathrm{for}\;q=1\;, \end{array} \end{array}$$

(55) $$\begin{array}{ccc} & & \exp_{q}\left(x\right)=\{\begin{array}{c} {\left(1+\left(1-q\right)\;x\right)}^{1/(1-q)}\;,\;\mathrm{for}\;0<q<2;\;q\neq 1\;,\\ \exp(x)\;,\;\mathrm{for}\;q=1\;. \end{array} \end{array}$$

In this case, the generalized product firstly introduced in [56,57] reads:

(56) $$\begin{array}{c} x⊗y={\left(x^{1-q}+y^{1-q}-1\right)}^{1/(1-q)}\;, \end{array}$$

whilst, the asymptotic approximation of $\ln_{q}\left(n{!}_{q}\right)$ obtained from (23) becomes:

(57) $$\begin{array}{c} \ln_{q}\left(n{!}_{q}\right)\approx \frac{n}{2-q}\;\left(\ln_{q}\left(n\right)-1\right)\;, \end{array}$$

where, in this case $\lambda_{q}=1$ and $e_{q}={\left(2-q\right)}^{1/(1-q)}$ are well defined in the range 0<q<2.

The *q*-version of the configurational entropy (28) is:

(58) $$\begin{array}{c} S_{2-q}^{B}\left[n\right]\approx \frac{n^{2-q}}{2-q}\;\frac{\sum_{i=1}^{\ell }{\left(\frac{n_{i}}{n}\right)}^{2-q}-1}{q-1}\;, \end{array}$$

and, posed $p_{i}=n_{i}/n$, it gives:

(59) $$\begin{array}{c} S_{2-q}^{B}\left[n\right]\approx \frac{n^{2-q}}{2-q}\;S_{2-q}^{S}\left[p\right]\;, \end{array}$$

which coincides with the result obtained firstly in [26].

To make contact with (38), we observe that, in this case, the function $I_{q}\left[p\right]$ (vs. $N_{q}\left(n\right)$) is related to the *q*-entropy $S_{q}^{S}\left[p\right]$ (vs. $S_{q}\left(n\right)$), according to:

(60) $$\begin{array}{c} I_{2-q}\left[p\right]=1+\frac{q-1}{2}\;S_{2-q}^{S}\left[p\right]\;, \end{array}$$

that is, in the Tsallis version of statistical mechanics, the functions $S_{q}$ and $I_{q}$ are not independent quantities. Therefore, in the framework of the *q*-deformed statistical mechanics the only *q*-version of the probability entropy seems to capture all relevant statistical information contained in the *q*-version of the configurational entropy.

By pushing relation (60) in (38), we promptly obtain relation (59).

## 6. Conclusions

With the aim of better understanding the emergence of generalized distributions often observed in natural and artificial complex systems, we have investigated a possible derivation of the configurational Boltzmann entropy by introducing a prescription for combinatorics dictated by an analytical function $f_{\left\{\pi \right\}}\left(x\right)$, dependent on a set of deformation parameters {π}, which takes into account possible statistical correlations among the monade of the system. A possible justification of the generalized multinomial coefficient may be derived from the presence of interactions between the constitutents of the system responsible for the changing of the particles allocation among the single-particle states available in the energy space. This fact consequently cause a change in the shape of its equilibrium distribution, different from the exponential one predicted by the Boltzmann entropy based on the usual multinomial coefficient. The latter is re-obtained in a suitable limit $\left\{\pi \right\}\to \left\{\pi_{0}\right\}$, where it is supposed that the interactions/correlations affecting the system are turned off so that, in the same limit, it is understood that the system reduces to an ensemble of ideal not interacting particles.

We sought, among the many possibilities, a generating function $f_{\left\{\pi \right\}}\left(x\right)$ by looking at an asymptotic approximation for the {π}-factorials of large numbers, that works in the same way as the Stirling approximation does for the standard factorial. In this way, the generating function is given by the solution of a simple differential–difference equation and the resulting configurational entropy (written in the occupational number representation) is the scaled version of the entropy of degree (κ,r) introduced about fifty years ago by Sharma–Taneja–Mittal in the framework of the information theory.

It is worth observing that generalized entropic forms consistent with power-law asymptotic distributions may also be related to certain fractional–differential equations or fractal–differential equations that differ substantially from the differential–difference equation used in this work. For instance, generalized entropies are derived within a kinetic approach based on a fractional diffusive equation [50] or in [58], where a study on fractal networks has been done within the Tsallis statistics.

More in general, the extension of statistical physics to the study of certain phenomenologies characterized by power-law distributions is usually non-trivial and may deal with entropic functions that differ substantially from those belonging to the Sharma–Taneja–Mittal family.

Finally, we explored the relationships between the generalized version of the Boltzmann configurational entropy and the corresponding version of the Shannon probability entropy showing that the known relation (4) acquires a rich structure in the generalized case [cfr. (38)]. In fact, if in the standard case all information contained in the configurational entropy is also carried out by the Shannon probability entropy, in the generalized case the only probability entropy of degree (κ,r) is not enough, in general, to capture all information contained in the corresponding configurational entropy. This is reflected by the new quantity $I_{\kappa ,\;r}\left[p\right]$, function of the probability distribution, which appears in relation (38).

## Figures and Tables

**Figure A1:**
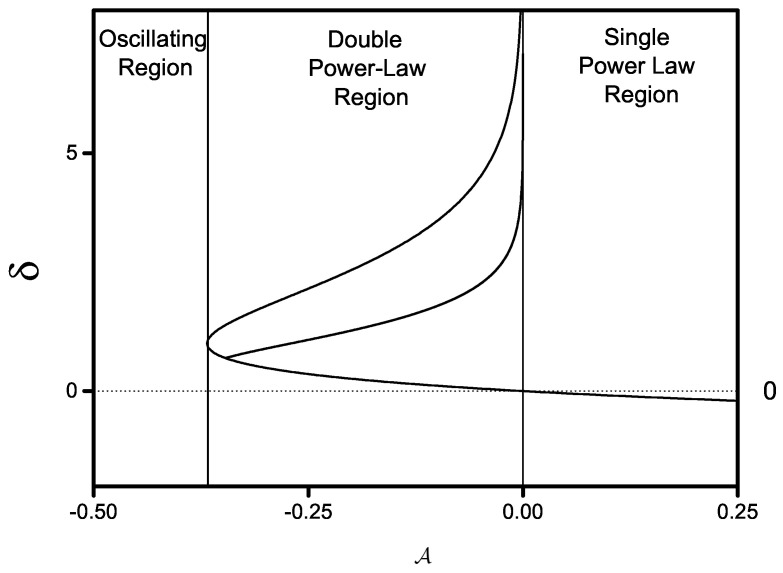
Representation, in the (A,δ)-parameters plane, of the solutions of the characteristic Equation (A11).

## Appendix A. Asymptotic Approximation of $\ln_{\left\{p\right\}}\left(n{!}_{\left\{p\right\}}\right)$

In this appendix, we derive an asymptotic approximation of the relation

(A1) $$\begin{array}{c} \ln_{\left\{p\right\}}\left(n{!}_{\left\{\pi \right\}}\right)=\sum_{i=1}^{n}\ln_{\left\{\pi \right\}}\left(i\right)\;, \end{array}$$

in the limit of large n for a special class of generalized logarithms whose generator function $f_{\left\{\pi \right\}}\left(x\right)$ is, in a sense, related to a delay equation.

As a first step, following [26,27], let us approximate the summation in (A1) by the integral

(A2) $$\begin{array}{c} \sum_{i=1}^{n}\ln_{\left\{\pi \right\}}\left(i\right)\approx \int_{0}^{n}\ln_{\left\{\pi \right\}}\left(x\right)\;dx=\int_{0}^{n}\ln\left(f_{\left\{\pi \right\}}\left(x\right)\right)\;dx\;. \end{array}$$

To handle this integral it is more convenient to introduce a new function $h_{\left\{\pi \right\}}\left(x\right)$ related to $f_{\left\{\pi \right\}}\left(x\right)$ in

(A3) $$\begin{array}{c} h_{\left\{\pi \right\}}\left(\ln(x)+\delta \right)=\ln\left(f_{\left\{\pi \right\}}\left(x\right)\right)\equiv \ln_{\left\{\pi \right\}}\left(x\right)\;, \end{array}$$

where $h_{\left\{\pi \right\}}\left(x\right)$ is a strictly monotonic increasing function for $x\in {ℜ}^{+}\to ℜ$ and δ an arbitrary constant that will be fixed in the sequel.

As a next step, let the function $h_{\left\{\pi \right\}}\left(x\right)$ be a solution of the following simple difference–differential equation,

(A4) $$\begin{array}{c} \frac{d\;y(x)}{dx}=\lambda \;y\left(x+\delta \right)-y\left(x\right)\;, \end{array}$$

with λ another constant.

It is straightforward to verify that if $h_{\left\{\pi \right\}}\left(x\right)$ fulfills (A4) than, from (A2), we have:

(A5) $$\begin{array}{ccc} \int_{0}^{n}\ln\left(f_{\left\{\pi \right\}}\left(x\right)\right)\;dx & = & \int_{0}^{n}h_{\left\{\pi \right\}}\left(\ln(x)+\delta \right)\;dx\\ & = & \frac{1}{\lambda }\int_{0}^{n}x\;\frac{d}{dx}h_{\left\{\pi \right\}}\left(\ln(x)\right)\;dx+\frac{1}{\lambda }\int_{0}^{n}h_{\left\{\pi \right\}}\left(\ln(x)\right)\;dx\;, \end{array}$$

and after an integration by part we obtain:

(A6) $$\begin{array}{c} \int_{0}^{n}h_{\left\{\pi \right\}}\left(\ln(x)+\delta \right)\;dx=\frac{n}{\lambda }\;h_{\left\{\pi \right\}}\left(\ln(n)\right)\;. \end{array}$$

Here, we assumed that a possible singularity in $h_{\left\{\pi \right\}}\left(x\right)$ for x→0 is mild enough to satisfy $\lim_{x\to 0}x\;h_{\left\{\pi \right\}}\left(x\right)=0$, so that (A6) can be finally rewritten in:

(A7) $$\begin{array}{c} \int_{0}^{n}\ln_{\left\{\pi \right\}}\left(x\right)\;dx=\frac{n}{\lambda }\;\ln_{\left\{\pi \right\}}\left(\frac{n}{\alpha }\right)\;, \end{array}$$

where $\alpha =e^{\delta }$.

Therefore, whenever the function $f_{\left\{\pi \right\}}\left(x\right)$ is related to the solutions of (A4) by means of the transformation (A3), the following asymptotic relation holds

(A8) $$\begin{array}{c} \ln_{\left\{\pi \right\}}\left(n{!}_{\left\{\pi \right\}}\right)\approx \frac{n}{\lambda }\;\ln_{\left\{\pi \right\}}\left(\frac{n}{\alpha }\right)\;. \end{array}$$

In the following we are interested to those solutions that capture the main properties of the standard logarithm, that is,

1. $\ln_{\left\{\pi \right\}}\left(1\right)=0$,
2. $d\ln_{\left\{\pi \right\}}\left(x\right)/dx>0$,
3. $d^{2}\ln_{\left\{\pi \right\}}\left(x\right)/dx^{2}<0$,

for $x\in {ℜ}^{+}\to ℜ$ and that reduces, in an opportune limit $\left\{\pi \right\}\to \left\{\pi_{0}\right\}$, to the classical logarithm: $\ln_{\left\{\pi_{0}\right\}}\left(x\right)=\ln\left(x\right)$.

Equation (A4) belongs to the family of the delay equations. Its general solution is given by:

(A9) $$\begin{array}{c} y\left(x\right)=\sum_{i}a_{i}\;y_{i}\left(x\right)\;, \end{array}$$

where $a_{i}$ are integration constants fixed by the initial conditions while $y_{i}$ are particular solutions of (A4) corresponding to eigenvalues of the associated characteristic equation:

(A10) $$\begin{array}{c} 1+\omega =\lambda \;e^{\delta \;\omega }\;, \end{array}$$

obtained from (A4) with the ansatz $y\left(x\right)=e^{\omega \;x}$.

Since the characteristic equation is transcendental, it has a countably infinite set of solutions. In the sequel, we limit our analysis only to the case of real eigenvalues so that the number of possible solutions may be 0, 1 or 2, depending by the constants λ and δ.

Rewriting (A10) in:

(A11) $$\begin{array}{c} \mu \;e^{\mu }=A\;, \end{array}$$

with μ=−δ(1+ω) and $A=-\lambda \;\delta \;e^{-\delta }$, it follows that for A≥0 there is just one single eigenvalue $\mu_{0}=W_{0}\left(A\right)$ where $W_{0}\left(x\right)$ is the principal branch of the Lambert function, such that:

(A12) $$\begin{array}{c} \omega_{0}=-1-\frac{1}{\delta }\;W_{0}\left(A\right)\;. \end{array}$$

This solution reduces to $\omega_{0}=-1$ for A=0.

Otherwise, for −1/e<A<0, Equation (A11) admits two distinct eigenvalues $\mu_{1}=W_{-1}\left(A\right)$ and $\mu_{2}=W_{0}\left(A\right)$, where $W_{-1}\left(x\right)$ is the −1-branch of the Lambert function, with:

(A13) $$\begin{array}{c} -\infty <\mu_{1}<-1\;,\;-1<\mu_{2}<0\;. \end{array}$$

They become coincident, $\mu_{1}=\mu_{2}=-1$, for A=−1/e.

Finally, for A<−1/e, (A11) has only complex conjugate eigenvalues.

Clearly, from (A3), solution (A12) gives:

(A14) $$\begin{array}{c} h_{\omega_{0}}\left(\ln(x)+\delta \right)=c\;x^{\omega_{0}}\;, \end{array}$$

which is a pure power-law. It does not reduce to a standard logarithmic function in any limit and thus is not relevant in our context. The same considerations apply for A<−1/e from which oscillating solutions would be obtained.

Differently, for −1/e<A<0 we have:

(A15) $$\begin{array}{c} \omega_{1}=\frac{1}{\delta }\;|W_{-1}\left(A\right)|-1\;,\;\mathrm{and}\;\omega_{2}=\frac{1}{\delta }\;\left|W_{0}\left(A\right)\right|-1\;, \end{array}$$

and, imposing (i), we obtain:

(A16) $$\begin{array}{c} h_{\omega_{1},\omega_{2}}\left(\ln(x)+\delta \right)=c\;\left(x^{\omega_{1}}-x^{\omega_{2}}\right)\;, \end{array}$$

where c>0 will be fixed in the sequel.

To satisfy (ii) we need $\omega_{1}\;\omega_{2}<0$ which requires, for fixed A∈[−1/e,0)

(A17) $$\begin{array}{c} |W_{0}\left(A\right)|<\delta <|W_{-1}\left(A\right)|\;,\;\mathrm{with}\;\lambda =-\frac{A}{\delta \;e^{-\delta }}\;, \end{array}$$

that, accounting for (iii), gives:

(A18) $$\begin{array}{c} 0<\omega_{1}<1\;,\;\mathrm{and}\;-1<\omega_{2}<0\;. \end{array}$$

In Figure A1, we depict, in the (A,δ)-parameters plane, the region corresponding to single real eigenvalue solution (single power-law region), two real eigenvalues (double power-law region) and complex conjugate eigenvalues (oscillating region) of the characteristic Equation (A11). The grey areas represent the region where $\omega_{1}\;\omega_{2}<0$. In particular, the dark grey area corresponds to the parameters region selected by the conditions (i)–(iii).

Finally, we choose the constant $c=\omega_{1}-\omega_{2}$ and, collecting all results, from (A3) we obtain:

(A19) $$\begin{array}{c} \ln_{\omega_{1},\omega_{2}}\left(x\right)=\frac{x^{\omega_{1}}-x^{\omega_{2}}}{\omega_{1}-\omega_{2}}\;, \end{array}$$

that is, the searched deformed logarithm. It reduces to the standard one in the $\left(\omega_{1},\;\omega_{2}\right)\to \left(0,\;0\right)$ limit.

To make contact with the existing literature [53], we re-define the deformation parameters $\omega_{1}$ and $\omega_{2}$ in

(A20) $$\begin{array}{c} \omega_{1}=r+\kappa \;,\;\omega_{2}=r-\kappa \;, \end{array}$$

to obtain the deformed logarithm in the form

(A21) $$\begin{array}{c} \ln_{\kappa ,r}\left(x\right)=\{\begin{array}{c} x^{r}\;\frac{x^{\kappa }-x^{-\kappa }}{2\;\kappa }\;,\;\mathrm{for}\;0<\kappa <1\;,\\ \ln(x)\;,\;\mathrm{for}\;\kappa =0. \end{array} \end{array}$$

In this parametrization, the allowed parameters region (A18) becomes: R={−|κ|<r<|κ|, if 0≤|κ|<1/2 and |κ|−1<r<1−|κ|, if 1/2≤|κ|<1}, while the scaling quantities $\lambda \equiv \lambda_{\kappa ,r}$ and $\alpha \equiv e_{\kappa ,r}$ in (A8) are given by

(A22) $$\begin{array}{ccc} & & \lambda_{\kappa ,r}=\{\begin{array}{c} \frac{{\left(1+r-\kappa \right)}^{(r+\kappa )/2\;\kappa }}{{\left(1+r+\kappa \right)}^{(r-\kappa )/2\;\kappa }}\;,\;\mathrm{for}\;0<\kappa <1\;,\\ 1\;,\;\mathrm{for}\;\kappa =0\;, \end{array} \end{array}$$

(A23) $$\begin{array}{ccc} & & e_{\kappa ,r}=\{\begin{array}{c} {\left(\frac{1+r+\kappa }{1+r-\kappa }\right)}^{1/2\;\kappa }\;,\;\mathrm{for}\;0<\kappa <1\;,\\ e\;,\;\mathrm{for}\;\kappa =0\;. \end{array} \end{array}$$

In the (κ,r)→(0,0) limit, $\lambda_{\kappa ,r}\to 1$ and $e_{\kappa ,r}\to e$, the Neperian number, and (A8) reduces to the well-known asymptotic approximation ln(n!)≈nln(n)−n, according to the Stirling formula.
